# Emotional reactivity and prosocial behaviour in response to witnessing social exclusion in adolescents with eating disorders and healthy controls

**DOI:** 10.1186/s40337-023-00927-4

**Published:** 2023-12-14

**Authors:** Katie Rowlands, Mima Simic, Janet Treasure, Valentina Cardi

**Affiliations:** 1 Department of Psychological Medicine, Institute of Psychiatry, Psychology and Neuroscience, King’s College London, London, UK; 2 Department of General Psychology, University of Padova, Padua, Italy; 3 South London and Maudsley NHS Foundation Trust, Maudsley Hospital, Denmark Hill, London, SE5 8AZ UK

**Keywords:** Eating disorders, Anorexia nervosa, Prosocial behaviour, Social skills, Adolescents, Negative emotion

## Abstract

**Background:**

Prosocial behaviour can promote positive social interactions and it is a key skill in adolescence. People with emotional problems or psychiatric disorders, such as people with eating disorders might have impairments in prosocial behaviour, due to broader documented difficulties in underlying processes (e.g., mentalizing).

**Methods:**

The aim of this study was to examine prosocial behaviour in adolescents with eating disorders compared to healthy controls, using a computerised behavioural task. Adolescents (*N* = 123) including patients with eating disorders (*n* = 61) and healthy adolescents (*n* = 62) played a four-player computerised Prosocial Cyberball Game with three pre-programmed avatar players. During the task, participants witnessed the exclusion of one of the players, and subsequently had the opportunity to compensate for this by throwing the ball more often to the excluded player. Throughout the game, participants rated the level of negative emotion in themselves and in the excluded player.

**Results:**

Patients made significantly fewer ball tosses towards the excluded player during the compensation round compared to healthy controls (large effect size). Patients reported a significantly smaller increase in negative emotion after witnessing the exclusion and a significantly smaller decrease in negative emotion following the compensation round (large effect sizes). Patients also estimated a smaller decrease in negative emotion in the excluded player following the compensation round (medium effect size). There were no significant associations between these outcomes and eating disorder psychopathology in patients.

**Conclusions:**

Compared to healthy adolescents, adolescent patients with eating disorders demonstrated less prosocial compensatory behaviour towards a computerised victim of social exclusion. In addition, they reported flatter negative emotion in themselves in response to witnessing and compensating for exclusion, and in the excluded player following compensation. If these findings are replicated, interventions to target these difficulties might contribute to improvements in social functioning in this patient group.

**Supplementary Information:**

The online version contains supplementary material available at 10.1186/s40337-023-00927-4.

## Abstract (plain language summary)

Prosocial behaviour (actions that benefit others) can promote positive social relationships. People with eating disorders may have impairments in prosocial behaviour because of difficulties in underlying processes (e.g., identifying emotions in oneself and in others). This study explored prosocial behaviour in 61 adolescents with eating disorders (77% anorexia nervosa) and 62 healthy adolescents, with an average age of 16. Each participant joined a 4-player computerised ball-tossing game with three pre-programmed avatars (i.e., only the participant was really playing the game; the three other avatars were not representing real players). Participants were initially included fairly in the game (i.e., the avatars were programmed to toss the ball equally to one another). In the next round, the participant merely observed the game (they could not actively participate). During this observation round, two of the avatars excluded the third avatar. In the final round, the participant was able to participate again, and could toss the ball to any of the three avatar players. Thus, the participant had the opportunity to compensate the avatar victim by tossing the ball more frequently to them. Throughout the game, the participant also rated the levels of negative emotion in themselves and in the avatar victim. After observing the exclusion, on average all participants tossed the ball more frequently to the avatar victim, but patients did so less frequently. Similarly, all participants reported more negative emotion, but this increase was smaller in patients. After the opportunity to compensate the victim, all participants reported less negative emotion in themselves and in the victim, but this decrease was smaller in patients. These outcomes were not linked to the severity of eating disorder symptoms in patients.

**Supplementary Information:**

The online version contains supplementary material available at 10.1186/s40337-023-00927-4.

## Highlights

- Patients made fewer ball-tosses to an excluded avatar after witnessing exclusion in a computerised task
- Patients reported a smaller increase in negative emotion after witnessing the exclusion
- Patients reported a smaller decrease in negative emotion after compensating the excluded player
- Patients estimated smaller decreases in negative emotion in the excluded player following compensation

**Supplementary Information:**

The online version contains supplementary material available at 10.1186/s40337-023-00927-4.

## Introduction

Prosocial behaviour is a multifaceted umbrella term which describes a range of voluntary behaviours that benefit others, such as cooperation, helping, giving, trust and reciprocity [1, 2]. These behaviours are particularly important during adolescence, a phase of individual development characterised by the challenge to fit in with groups of peers [3]. In adolescence, prosocial behaviour has been associated with more positive and less negative friendships [4], whereas longitudinal studies have identified bidirectional associations between prosocial behaviour and positive friendship quality [5], and between prosocial behaviour and lower peer rejection in adolescents [6].

Prosocial behaviour appears to be a protective factor for psychological wellbeing in adolescents [7]. In a meta-analysis of the literature, prosocial behaviours were significantly associated with lower internalising and externalising symptoms on average [8]. In an 11-year cohort study from childhood (age 3) through to adolescence (age 14), youth who displayed more early prosocial behaviours (as reported by parents in the Strength and Difficulties Questionnaire; [9]), tended to experience fewer emotional problems from age 5 to 14 years. Consistently, psychopathology experienced at early time points reduced the likelihood that participants engaged in prosocial behaviour at later time points [10]. In a recent systematic review, prosocial behaviour was identified as a protective factor for adolescent mental health following a period of social isolation due to the Covid-19 pandemic [11, 12]. These findings suggest that prosocial behaviour is generally associated with less emotional problems during adolescence.

Adolescents with difficulties in psychological processes that drive prosocial behaviour, such as mentalizing (understanding mental states of self and others; [13]), empathy (sharing emotions of others; [14–16]), and emotion regulation (ways of responding to emotional experiences; [17]) might struggle with prosocial behaviour. One study using the Prosocial Cyberball Game in a sample of Dutch adolescents (aged 9–17 years) demonstrated that those with higher levels of empathy compensated for the social exclusion of an unknown virtual peer by tossing the ball more frequently to that player after observing the unknown peer be excluded by the group, compared to the initial fair round of the game [18]. In line with these findings, in another study, 20 younger adolescents (aged 13 years), were scanned using functional magnetic resonance imaging (fMRI) whilst observing inclusion and exclusion of an unknown player during the Prosocial Cyberball Game [19]. Observing exclusion compared to inclusion activated brain regions involved in mentalizing, particularly among participants who reported high levels of empathy. Further, those who displayed more activity in affective, pain-related brain regions during the observed exclusion wrote more prosocial emails to the excluded victims afterwards, compared to observed inclusion rounds [19].

Few studies have investigated prosocial behaviour in adolescents with mental health difficulties. In a recent study [20] adolescents with eating disorders scored similarly on prosocial behaviours (as reported by parents in the Strength and Difficulties Questionnaire; 9), compared to adolescents with other psychiatric disorders or learning disabilities. These findings suggest that the psychopathology experienced by adolescents with eating disorders might interfere with prosocial behaviours, as indicated by the perspectives of parents [20]. However, given that no healthy control group was included, it is premature to conclude on potential problems with prosocial behaviour in this patient group.

Adolescents with eating disorders display a range of difficulties in social cognition processes that might explain possible problems with prosocial behaviour. These include difficulties recognising emotions in others [21], mentalisation (e.g., excessive inferring of mental states with a limited basis; [22]) theory of mind [23, 24], and in cognitive domains of empathy [14, 25]. Additional difficulties have been identified in emotional awareness and expression (e.g. reduced emotional awareness and inhibition of emotional expressions due to the numbing effect of the eating disorder symptoms; [26, 27]), and emotion regulation (e.g., greater use of maladaptive emotion regulation strategies and less use of adaptive strategies to cope with aversive emotional states) in adolescents with eating disorders compared to healthy adolescents [28]. These difficulties have been highlighted in both adolescents with anorexia nervosa and bulimia nervosa, although some differences have been identified between eating disorder sub-types (e.g., greater difficulties with impulse control, goal-directed behaviours, and access to effective emotion regulation strategies in adolescents with binge-purge episodes compared to those with restrictive symptoms only; [29]).

Considering these findings that patients with eating disorders have difficulties with skills involved in prosocial behaviour, the first aim of this study was to examine prosocial behaviour in patients with eating disorders compared to healthy controls. The hypothesis was that patients would show less prosocial behaviour, as demonstrated by fewer ball tosses towards the excluded player after witnessing the exclusion. Given the findings that *observing* exclusion typically leads to increases in negative emotion similar to that which is felt when experiencing exclusion [30–32], the second aim was to examine the impact of exclusion and compensation on the trajectory of negative emotion in patients compared to healthy controls. Based on these findings that patients display reduced emotional awareness and expression [26, 27], and greater use of maladaptive emotion regulation strategies [28], the hypothesis was that patients would report flatter negative emotion throughout the task. Similarly, the third aim was to examine changes in negative emotion in the excluded player in response to exclusion and compensation, as estimated by patients compared to healthy controls. Based on the evidence that patients with eating disorders demonstrate some difficulties with relevant social-emotional skills such as identifying emotions in others [21], the hypothesis was that patients would estimate flatter negative affect in the excluded player throughout the task. The final aim was to explore whether there were any associations between these outcomes and the severity of eating disorder psychopathology in patients. The hypothesis was that patients who showed less prosocial behaviour would report a greater severity of eating disorder psychopathology.

## Methods

### Participants

Adolescents aged 12–18, either with an eating disorder diagnosis (anorexia nervosa, bulimia nervosa, or meeting criteria for otherwise specified feeding or eating disorder), or with no history of psychiatric disorders were invited to participate in the study. In order to be eligible, participants also had to be fluent in English, with no severe visual impairments, neurological conditions or severe psychiatric comorbidities (e.g. psychosis, substance abuse). Patients were recruited from specialist eating disorder services and had received an eating disorder diagnosis from a psychiatrist, or they were recruited from the community via flyers and online advertisements. Participants who were recruited from the community self-reported their eating disorder diagnosis and were screened for eating disorders over the telephone by a trained researcher (KR), under the supervision of a qualified Clinical Psychologist (VC). The telephone screening involved completing the Structured Clinical Interview for DSM Disorders-Researcher Version (SCID-5-RV). Healthy controls were recruited from the community via flyers and online advertisements only, and were also screened for current/lifetime psychiatric disorders using the SCID-5-RV. All data were collected before the Covid-19 pandemic.

### Measures

#### Demographic and clinical characteristics

A demographic and clinical questionnaire was administered to all participants. The questionnaire consisted of items to assess the following variables: age, gender, weight, height, eating disorder diagnosis, psychiatric comorbidities, receipt of current clinical treatment, and use of psychiatric medications.

#### Eating Disorder Examination Questionnaire (EDE-Q; [33])

The “Eating Disorder Examination Questionnaire” (EDE-Q; [33]) is a 36-item self-report questionnaire to assess the severity of core eating disorder psychopathology in the past month. The global scale consists of four subscales, each assessing a specific domain of eating disorder psychopathology: eating concerns, shape concerns, weight concerns and dietary restraint. Scores are calculated for the total score and for each subscale independently. In this study, internal consistency was high for the total scale (*α* = .97) and the subscales restraint (*α* = .84), eating concerns (*α* = .87), shape concerns (*α* = .95), and weight concerns (*α* = .90).

#### Revised Children’s Anxiety and Depression Scale 25

In this study, The “Revised Children’s Anxiety and Depression Scale-25^′′^ (RCADS25; [34]) was used to characterise the patient sample in terms of comorbid anxiety and depression symptoms. It is a self-report questionnaire developed to assess symptoms of anxiety and depression in children and adolescents aged 8–18. The scale includes 25 items which are rated in terms of their frequency on a four-point scale ranging from never (0) to always (3). Typically, the questionnaire yields three scores, including a global score for anxiety and depression symptoms collectively, and subscale scores for anxiety and depression symptoms independently. In this study, internal consistency was high for the total score (*α* = .96) and anxiety (*α* = .92) and depression (*α* = .94) subscales.

#### The Prosocial Cyberball Game [18]

**Fig. 1:**
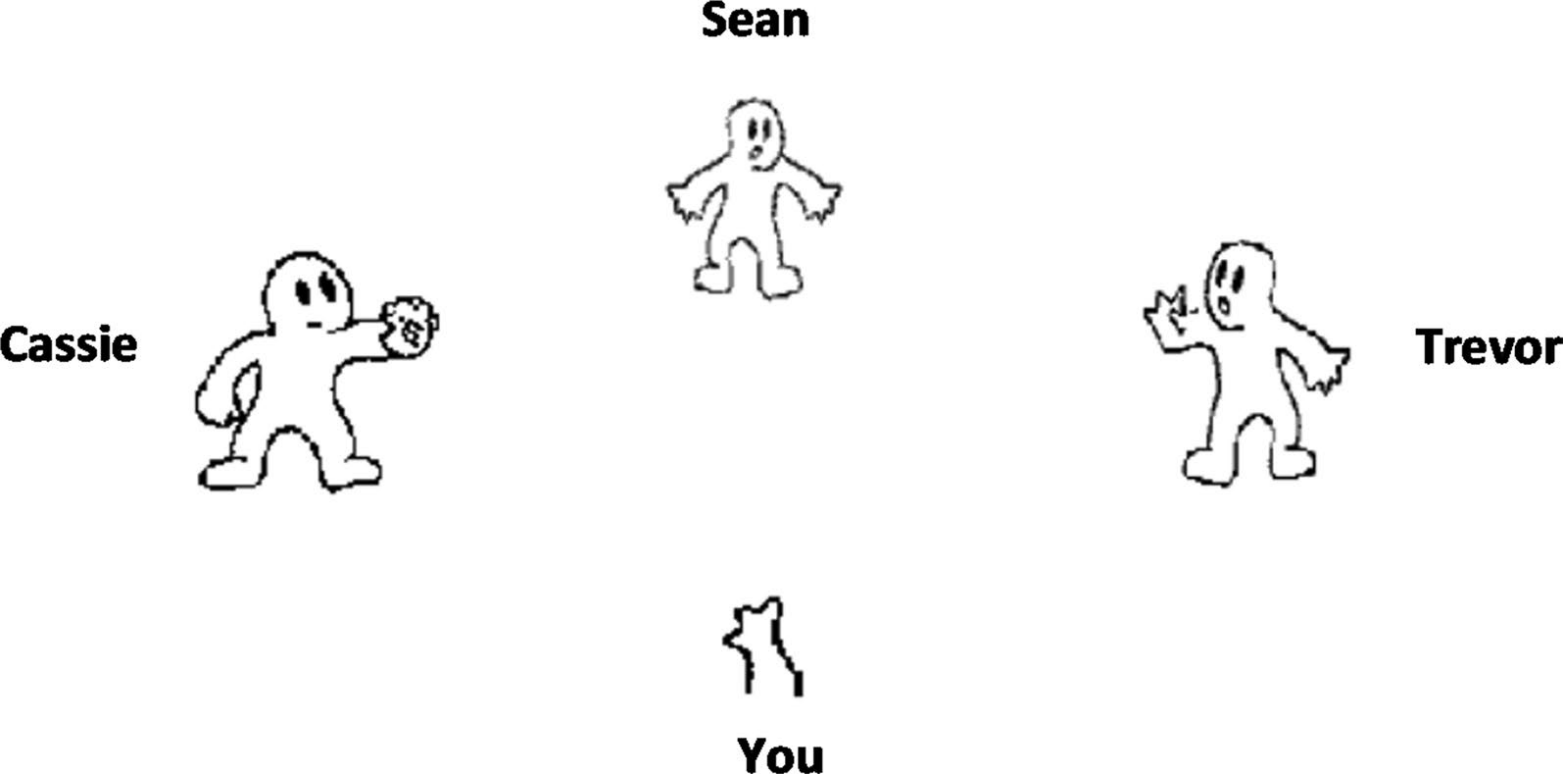
Screenshot of Prosocial Cyberball Game. Excluded player 4 = Sean

The Prosocial Cyberball Game [18] is a computerised task, which can be performed on a desktop computer or a laptop with either a hand-held mouse or touchpad. In this study, the task was hosted on the Inquisit web platform [35]. The task involved four players (three computer-generated players, players 1, 3, and 4, and the participant, player 2), who were all positioned facing each other in the middle top, bottom, left and right of the screen (see Fig. 1). In this study, the game consisted of three rounds.

The first round was the "Inclusion" round, which consisted of 48 trials. In this round, each player received the ball roughly the same number of times (12 times). The next round of the game was the “observation” round. In this round, the participant was instructed to simply observe (and not join in with) the game. This round consisted of 20 trials. During this round, players 1 and 3 excluded player 4, by never passing the ball to player 4. The final round was the “compensation” round. It consists of 48 trials. In this round, the participant (player 2) was re-integrated into the game and was thus able to throw the ball to any of the three players. However, players 1 and 3 continued to exclude player 4 by only passing the ball between one another and the participant. Following the observation and compensation rounds, participants reported on whether the game was fair or not (i.e. by simply selecting “yes” or “no”). If participants answer “no” they were asked to select the reason why the game was unfair. They could select the answer “because I received the ball less than the others” or “because someone else received the ball less than the others”. If the participant noticed that someone else received the ball less than others, they were asked to identify the excluded player.

Following each round of the game, participants rated the extent to which they experienced negative emotions (bad, unfriendly, angry, sad; [36]), on a scale from 1 (not at all) to 5 (extremely). Following the observation and compensation rounds, participants also estimated the extent to which the excluded player experienced the same negative emotions on the same rating scale (if the participant identified the exclusion).

### Procedure

Participants completed the Prosocial Cyberball task and clinical questionnaires at the research laboratory (Centre for Eating and Weight Disorders, King’s College London), in hospital, or from a computer at home, with the support of the researcher. The measures were completed as part of a larger project investigating interpersonal functioning in adolescents with eating disorders (some data from this project has been published previously; [37]). Weight and height were measured by the researcher, obtained from the clinical service or self-reported. Participants were given verbal and written instructions for each task.

### Statistical analyses

An independent t-test was used to examine the number of balls tossed to the excluded player in the compensation round in participants with eating disorders and healthy controls. According to the Shapiro–wilk test, the assumption of normality of data was violated for negative emotion in self at baseline and post-observation, and as estimated in the excluded player at post-observation and post-compensation. Repeated measures ANOVA, which is robust against violations of normality [38], was used to analyse these data. As there were no significant changes in negative emotion between the baseline and post-inclusion measurement time points, only the baseline negative emotion score was used as the ‘pre-exclusion’ time point in the ANOVAs. To examine changes in negative emotion in ‘self’ (the participant) after observing the exclusion, a repeated measures ANOVA was conducted with participant group as the independent variable and overall negative emotion as the dependent variable, with two levels (baseline and post-observation). To examine changes in negative emotion in the participant after having the opportunity to compensate for the excluded player, a second repeated measures ANOVA was conducted with participant group as the independent variable and negative emotion as the dependent variable with two levels (post-observation and post-compensation). To examine changes in perceived negative emotion in the excluded player, a third repeated measures ANOVA was conducted with participant group as the independent variable and negative emotion in the excluded player as the dependent variable with two levels (post-observation and post-compensation). Bivariate correlations were used to examine whether there were associations between prosocial behaviour, or changes in negative emotion ratings during the task (in the self or excluded player) and eating disorder psychopathology in patients (i.e., self-reported eating disorder symptoms over the past month as measured via the EDE-Q total score).

## Results

### Demographic and clinical characteristics

One-hundred and forty-five participants were enrolled in the study (for a flowchart of participation, see Additional file 1: Fig S1). Participants were excluded from the analyses and if they had incomplete data (*n* = 16), thus only those with complete data (*n* = 129) were included. Additional participants were excluded if they did not pass the manipulation check at post-observation (*n* = 6; see ‘Manipulation check’ section below). Thus, the final sample consisted of 123 participants, including 61 participants with eating disorders and 62 healthy controls.

There was no significant difference in age between participants with eating disorders (*M* = 16.13, *SD* = 1.15) compared to healthy controls (*M* = 15.97, *SD* = 1.17), *t*(121) = 0.781, *p* = .436. In the eating disorder group, there were 59 girls (97%) and two boys (3%). In the healthy control group, there were 40 girls (65%) and 22 boys (35%). This difference was significant (*p* < .001, Phi =  − .406; Fisher’s Exact Test). As expected, patients had a lower percentage median BMI (*M* = 93.21, SD = 10.83) compared to healthy controls (*M* = 112.44, *SD* = 24.43). This difference was significant, *t(84*) =  − 5.657, *p* < .001. Most patients had a diagnosis of anorexia nervosa (*n* = 47, 77%), with fewer cases of bulimia nervosa (*n* = 8, 13%) and eating disorder not otherwise specified (EDNOS, *n* = 6, 10%). Fifty-three patients (87%) were receiving treatment, including outpatient (*n* = 36, 59%), or inpatient/intensive day care (*n* = 17, 28%), and 30 (49%) patients reported taking psychiatric medications, including anti-depressants, anti-anxiety or antipsychotic medications (eight participants reported taking more than one type of medication). On the EDE-Q, 28 (46%) participants with eating disorders scored above 4, a commonly used cut-off for clinical range [33]. On the RCADS25, 28 (52%) patients scored in the clinical range (≥ 70) for depression symptoms, 25 (46%) for anxiety symptoms, and 29 (48%) for the total score (comorbid anxiety and depression symptoms). No participants in the healthy control group scored in the clinical ranges on the EDE-Q or RCADS25.

### Manipulation check

As expected, following the observation round, almost all participants (*n* = 128, 99%) correctly rated the game they observed as “unfair” (one participant with an eating disorder rated the game as “fair” and was excluded from the analysis). Most of these participants (*n* = 123, 95%) correctly attributed the unfairness to the exclusion of player 4 (five healthy controls did not report noticing the exclusion and were also excluded from the analysis). Thus, in total, six participants were excluded from the analysis due to manipulation check failure. Interestingly, following the compensation round, significantly more participants with eating disorders (*n* = 30, 50%) rated the game as “unfair” compared to healthy controls (*n* = 16, 26%), χ2 = 7.599, *p* = .006. Most of these participants (*n* = 45, 98%) correctly attributed this to the exclusion of player 4 (one participant with an eating disorder incorrectly attributed this to being excluded themselves). Only the participants who correctly rated the compensation round as ‘unfair’, due to the exclusion of player 4, received the post-compensation measures of negative mood in self, and the excluded player (for a flowchart of participation, see Additional file 1: Fig. S1).

### Prosocial behaviour

As shown in Table 1, patients made significantly fewer ball-tosses to excluded player 4 compared to healthy controls during the compensation round (large effect size). When boys were excluded from the analysis, this difference remained but became non-significant (large effect size).

**Table 1:** Ball-tosses towards excluded player in the Prosocial Cyberball Game

Dependent variable	ED *n* = 61 M (SD)	HC *n* = 62 M (SD)	*t*	*df*	*p*	*d*	*CI*
Ball-tosses to excluded player (compensation)	8.02 (1.64)	8.94 (2.52)	− 2.406	105	.018*	2.13	− .789 to − .074

Dependent variable	ED girls *n* = 59 M (SD)	HC girls *n* = 40 M (SD)	*t*	*df*	*p*	*d*	*CI*
Ball-tosses to excluded player (compensation)	7.98 (1.66)	8.50 (2.31)	− 1.298	97	.197	1.95	− .668 to .138

This table displays results from an independent samples t-test using raw number of ball-tosses towards the excluded player in the compensation round of the Prosocial Cyberball Game. Single asterik (*) indicates statistical significance at *p* < .05

### Ratings of negative emotion in self

As shown in Table 2, patients reported a significantly smaller increase in overall negative emotion in themselves between baseline and post-observation compared to healthy controls (large effect size), controlling for gender and percentage median BMI. This finding suggests that patients showed less negative emotion reactivity to observing exclusion. Patients also reported significantly smaller decreases in negative emotion between the post-observation and post-compensation rounds compared to healthy controls (large effect size). This finding indicates that the level of negative emotion triggered by witnessing the exclusion of player 4 remained for longer in patients. Results for each negative emotion individually are reported in Additional file 1: Table S1.

**Table 2:** Changes in negative emotion in self and excluded player at baseline, post-exclusion, post-observation and post-compensation, controlling for gender and percentage median BMI

Dependent variable	ED *n* = 61 M* (SD)*	HC *n* = 62 M* (SD)*	Factor(s)	*t*	*df*	*p*	ηp2
Negative emotion (self) baseline	2.12 (.89)	1.27 (.52)	Time	9.857	1,122	.002*	.075
Negative emotion (self) post-observation	3.32 (1.01)	3.40 (.83)	Time × Group	22.342	1,122	< .001**	.155
			Group	6.476	1,122	.012*	.050
			Time × Gender	.029	1,122	.864	.000
			Time × Percentage median BMI	.336	1,122	.563	.003

Dependent variable	ED *n* = 31 M* (SD)*	HC *n* = 14 M* (SD)*	Factor(s)	*t*	*df*	*p*	ηp2
Negative emotion (self) post-observation	3.39 (.95)	3.48 (.62)	Time	3.548	1, 41	.067	.080
Negative emotion (self) post-compensation	3.05 (.95)	2.71 (.80)	Time × Group	7.314	1, 41	.010*	.151
			Group	.100	1, 41	.753	.002
			Time × Gender	.802	1, 41	.376	.019
			Time × Percentage median BMI	.609	1, 41	.440	.015

Dependent variable	ED *n* = 29 M* (SD)*	HC *n* = 14 M* (SD)*	Factor(s)	*t*	*df*	*p*	ηp2
Estimated negative emotion (in excluded player) post-observation	3.80 (.82)	4.11 (.49)	Time	.796	1, 39	.378	.020
Estimated negative emotion (in excluded player) post-compensation	3.34 (.86)	3.29 (.85)	Time × Group	3.194	1, 39	.082	.076
			Group	.043	1, 39	.836	.001
			Time × Gender	.003	1, 39	.956	.000
			Time × Percentage median BMI	.775	1, 39	.384	.019

Negative emotion is the sum of scores for all negative emotions (feeling bad, sad, angry, and unfriendly), rated on a scale from 1 (not at all) to 5 (extremely). Single asterik (*) indicates statistical significance at *p* < .05. Double asterik (**) indicates statistical significance at *p* < .001

### Ratings of negative emotion in excluded player

As shown in Table 2, patients who rated the game as ‘unfair’ post-compensation estimated a smaller decrease in negative emotion in the excluded player between observation and compensation rounds compared to the healthy controls, controlling for gender and percentage median BMI. This difference showed a trend towards significance, with a medium effect size. This finding suggests that patients estimated less improvement in negative emotion in the excluded player following compensation for the exclusion. Results for each negative emotion individually are reported in Additional file 1: Table S1.

### Correlations with the severity of eating disorder psychopathology

In the eating disorder group, there was no significant correlation between the number of balls thrown to the excluded player in the compensation round and the severity of self-reported eating disorder psychopathology (*r* =  − .089, *p* = .497). There was also no significant correlation between the change in negative emotion (in self) between baseline and post-observation and eating disorder psychopathology (*r* =  − .125, *p* = .338). There was no significant correlation between patients’ estimation of change in negative emotion in the excluded player between post-observation and post-compensation and eating disorder psychopathology (*r* = .073, *p* = .705).

## Discussion

The overall aim of this study was to examine the impact of witnessing social exclusion on prosocial behaviour towards an excluded player, negative emotion in the self, and perceptions of negative emotion in the excluded player, in adolescent patients with eating disorders compared to healthy controls, using a computerised behavioural task. In line with the first three hypotheses, patients tossed the ball to the excluded player less frequently than the healthy control group in the compensation round. Patients also reported a smaller increase in negative emotion in themselves after witnessing the exclusion compared to healthy controls. After the observation round, both patients and healthy controls perceived an increase in negative emotion in the excluded player, and there was no significant difference in these ratings between groups. However, of those who noticed the exclusion in the compensation round, patients estimated a smaller decrease in negative emotion in the excluded player following the compensation round compared to healthy controls. In contrast to the final hypothesis, there were no associations between these outcomes and the severity of eating disorder psychopathology in the patient group.

The finding of lower prosocial behaviour in patients aligns with studies indicating more difficulties with interpersonal processes that drive prosocial behaviour in adolescents with eating disorders, such as mentalising [21, 39] and corroborate parent-reports of possible impairments in prosocial behaviour in adolescents with eating disorders [20]. Whilst the gender imbalance between the groups may have contributed to this finding, the effect size remained large in the analysis when only the girls were included. This provides a positive indication that the difference may be explained predominantly by participant group.

The flatter negative emotion reported by patients throughout the task might reflect the numbing effect that eating disorder symptoms can have on emotional experience, such that symptoms may function in part to help individuals avoid aversive emotional states [40]. Indeed, patients with disorders of over-control such as anorexia nervosa demonstrate a pervasive inhibition of emotional expression and low emotional awareness [26, 27]. These difficulties can manifest as incongruent expressions of emotion, such as consistent under-reporting of distress [41–43]. Similarly, these findings might reflect the evidence that patients’ with anorexia nervosa display a greater use of ‘maladaptive’ emotion regulation strategies to cope with aversive emotional states such as emotion suppression, characterised by attempts to decrease the expression of negative emotion when emotionally aroused [29, 44–48].

The finding that patients tended to estimate a smaller decrease in negative emotion in the excluded player following compensation, compared to healthy controls, might reflect broader underlying difficulties in recognising emotions in others [21], mentalisation [22], theory of mind [23, 24], or in cognitive domains of empathy [14, 25]. One alternative explanation could be that patients perceived the compensation itself to be less adequate at reducing negative emotion in the excluded player compared to healthy controls. Accordingly, a greater number of patients rated the game as unfair following the compensation round compared to healthy controls. However, as these psychological processes were not directly measured in this study, it is not possible to explain this finding definitively.

The lack of association between prosocial behaviour towards the excluded player, changes in negative emotion, as estimated in the self and the excluded player, and core eating disorder psychopathology in patients might indicate that the tendencies to express less negative emotion and compensate less in response to witnessing exclusion are more associated with unmeasured underlying social-emotional skills (e.g. emotional expression, emotion regulation, mentalizing, empathy), rather than eating disorder symptoms specifically. However, this finding needs to be replicated in larger studies.

## Strengths and limitations

The main strength of this study is the use of a behavioural task to assess prosocial behaviour, given that this behaviour is typically measured using questionnaires in adolescents which are subject to limitations such as social desirability bias, and use hypothetical scenarios [49]. Another strength is the study design, which facilitated the comparison of a clinical group of patients with eating disorders and a healthy control group. The key limitation of this study is the omission of measures of psychological processes related to prosocial behaviour such as empathy, mentalizing, and use of emotion regulation strategies, as well as data on comorbidities such as autistic symptoms. The use of these assessments in conjunction with behavioural measures of prosocial behaviour might provide further insight into whether specific underlying processes might help to explain the subtle yet meaningful differences in outcomes of the Prosocial Cyberball task between adolescents with eating disorders and healthy controls. An additional limitation was that the post-compensation measures of negative mood in self and excluded player were only administered to participants who reported the game was still unfair post-compensation. Collecting these ratings from all participants would allow comparison of changes in negative mood between those who rated the game as ‘fair’ and those who rated the game as ‘unfair’ post-compensation. These data could provide a more explicit indication as to why patients who rated the game as ‘unfair’ reported smaller changes in negative emotion in themselves and in the excluded player post-compensation compared to healthy controls.

## Conclusions

Compared to healthy controls, adolescent patients with eating disorders displayed lower prosocial behaviour and flatter negative emotion in response to witnessing and compensating for social exclusion compared to healthy controls. Patients also estimated flatter negative emotion in an excluded computerised player after the opportunity to compensate this player with prosocial behaviour. If replicated, these findings may inform targeted interventions to improve social functioning in this patient group.

### Supplementary Information

**Additional file 1. Table 1.** Changes in individual negative emotions in self and excluded player at baseline, post-exclusion, post-observation and post-compensation, controlling for gender and percentage median BMI. **Figure 1.** Flowchart of Participation in the Prosocial Cyberball Game.

## Data Availability

The dataset analysed during the current study is available from the corresponding author upon reasonable request.

## Abbreviations

RCADS25: Revised Children’s Anxiety and Depression Scale-Short Version
EDE-Q: Eating Disorder Examination Questionnaire
fMRI: Functional magnetic resonance imaging
SCID-5-RV: Structured Clinical Interview for DSM Disorders-Researcher Version
