# Supplementary material for: Emotional reactivity and prosocial behaviour in response to witnessing social exclusion in adolescents with eating disorders and healthy controls
Source: J Eat Disord. 2023 Dec 14;11:224. doi: 10.1186/s40337-023-00927-4 (PMC10722719; doi:10.1186/s40337-023-00927-4)
Supplement: Supplementary file 1 — Additional file 1. Table 1. Changes in individual negative emotions in self and excluded player at baseline, post-exclusion, post-observation and post-compensation, controlling for gender and percentage median BMI. Figure 1. Flowchart of Participation in the Prosocial Cyberball Game. [file 40337_2023_927_MOESM1_ESM.docx]

**Supplementary materials**

**Supplementary Table 1.**

Changes in individual negative emotions in self and excluded player at baseline, post-exclusion, post-observation and post-compensation, controlling for gender and percentage median BMI.

| Dependent variable | ED = 61 M (SD) | HC = 62 M (SD) | Factor(s) | *df* | *F* | *p* | ηp2 |
| --- | --- | --- | --- | --- | --- | --- | --- |
| Feeling ‘bad’ (self) baseline | 2.54 (1.15) | 1.26 (.55) | Time | 1, 118 | 5.600 | .020* | .045 |
| Feeling ‘bad’ (self) post-observation | 3.31 (1.25) | 3.54 (1.03) | Time x Group | 1, 118 | 26.940 | < .001** | .186 |
|  |  |  | Group | 1, 118 | 6.754 | .011* | .054 |
|  |  |  | Time x Gender | 1, 118 | .743 | .390 | .006 |
|  |  |  | Time x Percentage median BMI | 1, 118 | .000 | .989 | .000 |
| Feeling ‘sad’ (self) baseline | 2.23 (1.24) | 1.30 (.760) | Time | 1, 118 | 1.261 | .264 | .011 |
| Feeling ‘sad’ (self) post-observation | 3.46 (1.31) | 3.15 (1.15) | Time x Group | 1, 118 | 7.074 | .009* | .057 |
|  |  |  | Group | 1, 118 | 10.949 | < .001** | 085 |
|  |  |  | Time x Gender | 1, 118 | 1.310 | .255 | .011 |
|  |  |  | Time x Percentage median BMI | 1, 118 | .081 | .777 | .001 |
| Feeling ‘angry’ (self) baseline | 1.70 (.97) | 1.33 (.75) | Time | 1, 118 | 4.867 | .029* | .040 |
| Feeling ‘angry’ (self) post-observation | 3.44 (1.27) | 3.43 (1.13) | Time x Group | 1, 118 | 4.493 | .036* | .037 |
|  |  |  | Group | 1, 118 | 1.750 | .188 | .015 |
|  |  |  | Time x Gender | 1, 118 | 1.194 | .277 | .010 |
|  |  |  | Time x Percentage median BMI | 1, 118 | .973 | .326 | .008 |
| Feeling ‘unfriendly’ (self) baseline | 1.97 (1.05) | 1.21 (.45) | Time | 1, 118 | 8.058 | .005* | .064 |
| Feeling ‘unfriendly’ (self) post-observation | 3.05 (1.38) | 3.49 (1.10) | Time x Group | 1, 118 | 15.031 | < .001** | .113 |
|  |  |  | Group | 1, 118 | .783 | .378 | .007 |
|  |  |  | Time x Gender | 1, 118 | .713 | .400 | .006 |
|  |  |  | Time x Percentage median BMI | 1, 118 | .524 | .470 | .004 |
| Dependent variable | ED = 29 | HC = 15 | Factor(s) | *df* | *F* | *p* | ηp2 |
| Feeling ‘bad’ (excluded player) post-observation | 4.14 (.99) | 4.33 (.49) | Time | 1, 40 | .081 | .777 | .002 |
| Feeling ‘bad’ (excluded player) post-compensation | 3.41 (1.15) | 3.47 (.64) | Time x Group | 1, 40 | .166 | .686 | .004 |
|  |  |  | Group | 1, 40 | .089 | .767 | .002 |
|  |  |  | Time x Gender | 1, 40 | .068 | .796 | .002 |
|  |  |  | Time x Percentage median BMI | 1, 40 | .000 | 1.000 | .000 |
| Feeling ‘sad’ (excluded player) post-observation | 4.21 (.82) | 4.07 (.83) | Time | 1, 39 | .176 | .677 | .004 |
| Feeling ‘sad’ (excluded player) post-compensation | 3.59 (1.05) | 3.21 (1.19) | Time x Group | 1, 39 | .861 | .359 | .022 |
|  |  |  | Group | 1, 39 | 1.704 | .199 | .042 |
|  |  |  | Time x Gender | 1, 39 | .215 | .645 | .005 |
|  |  |  | Time x Percentage median BMI | 1, 39 | .215 | .645 | .005 |
| Feeling ‘angry’ (excluded player) post-observation | 3.59 (1.21) | 4.00 (.96) | Time | 1, 39 | 3.932 | .054 | .092 |
| Feeling ‘angry’ (excluded player) post-compensation | 3.34 (1.11) | 3.21 (1.05) | Time x Group | 1, 39 | 5.688 | .022* | .127 |
|  |  |  | Group | 1, 39 | .273 | .604 | .007 |
|  |  |  | Time x Gender | 1, 39 | .093 | .762 | .002 |
|  |  |  | Time x Percentage median BMI | 1, 39 | 3.499 | .069 | .082 |
| Feeling ‘unfriendly’ (excluded player) post-observation | 3.34 (1.32) | 4.13 (.92) | Time | 1, 40 | .622 | .435 | .015 |
| Feeling ‘unfriendly’ (excluded player) post-compensation | 3.20 (1.29) | 3.33 (.98) | Time x Group | 1, 40 | 1.644 | .207 | .039 |
|  |  |  | Group | 1, 40 | .911 | .346 | .022 |
|  |  |  | Time x Gender | 1, 40 | .210 | .649 | .005 |
|  |  |  | Time x Percentage median BMI | 1, 40 | .081 | .778 | .002 |

Note. *Asterik (*) indicates significance at p < .05. ** at p < .001.*


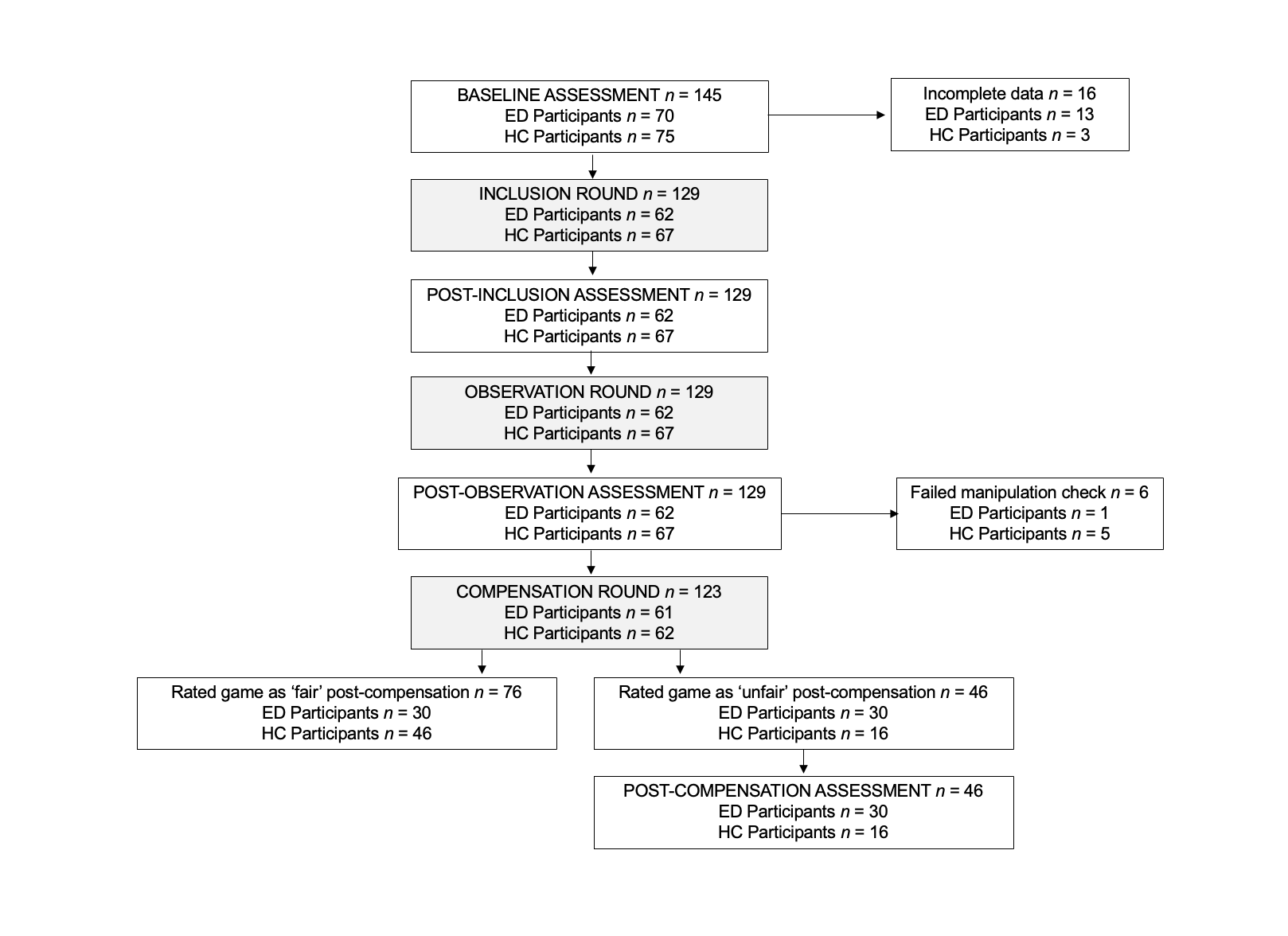


**Supplementary Figure 1.** Flowchart of Participation in the Prosocial Cyberball Game
